# Adefovir dipivoxil inhibits APL progression through degradation of the oncoprotein PML-RARA

**DOI:** 10.1186/s40164-022-00355-1

**Published:** 2022-11-20

**Authors:** Xubo Gong, Piaoping Kong, Teng Yu, Xibin Xiao, Lin Wang, Yiwen Sang, Xiang Li, Bin Zhang, Zhihua Tao, Weiwei Liu

**Affiliations:** 1 Department of Clinical Laboratory, The Second Affiliated Hospital, Zhejiang University School of Medicine, 88 Jiefang Road, Hangzhou, 310000 Zheijang People’s Republic of China; 2 Department of Hematology, The Second Affiliated Hospital, Zhejiang University School of Medicine, Hangzhou, 310000 Zheijang People’s Republic of China; 3 Department of Hematological Malignancies Translational Science, Gehr Family Center for Leukemia Research, Hematologic Malignancies and Stem Cell Transplantation Institute, Beckman Research Institute, City of Hope Medical Center, Duarte, CA USA

**Keywords:** Adefovir dipivoxil, Entecavir, APL, TRIB3

## Abstract

Acute promyelocytic leukemia (APL) is highly aggressive and is frequently associated with disseminated intravascular coagulation and high early death rates. Although all-trans retinoic acid (RA) induces complete remission in a high proportion of patients with APL, there are limited treatments for APL patients with RA resistance. Here we report an atypical APL patient, with an APL-like disease that developed very slowly without anti-leukemia therapy for nearly 2 years. During that time, the patient only intermittently received anti-HBV drugs, i.e., the combination of adefovir dipivoxil (ADV) and entecavir (ETV), leading us to hypothesize that ADV and/or ETV could inhibit APL progression. Our results showed that anti-HBV drugs ADV and ETV both exhibited significantly inhibitory effects on APL cells, and ADV indicated a much greater cytotoxic effect than ETV on APL cells. We further found that ADV significantly promoted APL cell differentiation and apoptosis, thereby restraining the progression of APL. Most importantly, our study uncovered a novel mechanism of ADV prohibiting APL progression, which was mediated, at least in part, by inhibition of TRIB3 and degradation of the oncoprotein PML-RARA, therefore leading to APL cell differentiation and apoptosis. Taken together, our study demonstrated that ADV, an anti-HBV drug, had significantly inhibitory effects on APL, and provided a novel therapeutic strategy for APL patients with RA resistance.

**Supplementary Information:**

The online version contains supplementary material available at 10.1186/s40164-022-00355-1.

## To the editor,

Acute promyelocytic leukemia (APL) is highly aggressive, and the disease may occur abruptly, with a relatively high early death rate (up to 30%) associated with coagulopathy [1–3]. Here we report an atypical APL case. The patient, treated with anti-HBV drugs, showed a very slowly progressing APL-like disease. Since this patient had never been treated with all-trans retinoic acid or chemotherapy, we hypothesized that anti-HBV drugs, i.e., adefovir dipivoxil (ADV) and/or entecavir (ETV), could inhibit APL progression. Our results showed that ADV exhibited significantly inhibitory effects on APL.

**Fig. 1:**
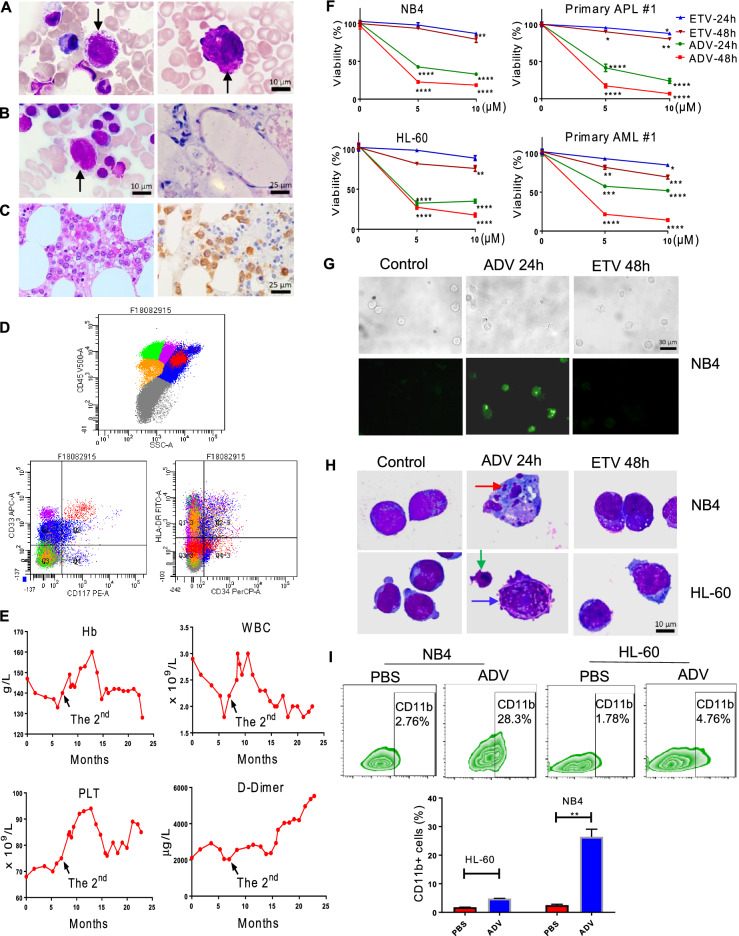
The clinical features of the specific patient, and the viability, apoptosis, and differentiation analysis of ADV to AML cells in vitro. **A** The first BM examination showed some abnormal promyelocytes (9.0%), with large granules and bundles of Auer rods (black arrow). **B** The second BM examination revealed 11.0% of abnormal promyelocytes (black arrow) on BM smears, and decreased BM hyperplasia. **C** The third BM examination showed increased abnormal promyelocytes on BM biopsies, which were strongly positive for myeloperoxidase. **D** Flow cytometry of the second BM examination showed a low proportion of abnormal myeloid cells (1.5%) within the gate defined by side scatter/ CD45 expression (red marker), which expressed myeloid markers CD117, CD33, rather than hematopoietic stem marker CD34 or human leukocyte antigen-DR isotype (HLA-DR). **E** The patient’s complete blood count and d-Dimer from the first to the third BM examinations, with a period more than 22 months. After 20 months since the first BM examination, d-Dimer increased obviously while hemoglobin decreased rapidly. The 2nd, the second BM examination. Hb, hemoglobin. WBC, White blood cells. PLT, platelet. **F** The viabilities of NB4, HL-60, primary APL and AML cells were examined with CCK-8 assay after treatment with 0, 5 and 10 µM ADV or ETV for 24 and 48 h. The graphs show cells proliferation relative to DMSO (cells treated with DMSO alone). **G** After treatment of 5 µM ADV for 24 h, it induced the apoptosis of NB4 cells with TUNEL assay. But the apoptosis was not increased in presence of 5 µM ETV for 48 h compared with control group (NB4 cells treated with DMSO alone). Scale bar, 25 μm. **H** With Wright-Giemsa staining, the typical apoptotic cells were easily found in presence of 5 µM ADV for 24 h, including condensed nuclear chromatin, nuclei shrinkage (blue arrow), nuclear fragmentation (green arrow) and apoptotic bodies (red arrow). Scale bar, 10 μm. **I** NB4 and HL60 cell were treated with 0.25 µM ADV for 96 h, increased expression levels of myeloid surface markers CD11b, especially on NB4 cells, were observed using flow cytometer

The patient had a long history of hepatitis B virus (HBV) infection for more than 40 years. Since 2017, the patient had been treated with anti HBV drugs intermittently. The 1st bone marrow (BM) examination was performed due to low white blood cells and platelets, and BM smears showed 9.0% abnormal promyelocytes, bundles of Auer rods were present (Fig. 1A). The patient was not given anti-leukemia therapy. The 2nd BM examination was performed 7 months later, and BM smears showed 11.0% abnormal promyelocytes and BM biopsies revealed decreased BM hyperplasia (Fig. 1B). Flow cytometry showed a low proportion of abnormal myeloid cells (1.5%) within CD45+ cells, which also expressed CD9, CD13, CD33, CD38, CD64, CD117, CD123, but not CD15, CD34, or HLA-DR (Fig. 1D). Cytogenetic analysis of BM cells revealed 46, XY, and the Bcr1 type of *PML-RARA* fusion was negative.

The patient had been monitored for another 15 months without anti-leukemia therapy, and then the 3rd BM examination was performed. Abnormal promyelocytes were increased on BM smears (34.0%) and biopsies (Fig. 1C, E). It showed a Bcr3 type of *PML-RARA*. Therefore, at this time, the patient was diagnosed as APL with *PML-RARA* (Additional file 1: Table S1). After treatment with all-trans-retinoic-acid and arsenic trioxide, the patient achieved complete remission.

APL, without therapy, progresses quickly with a high risk of mortality. It is very unusual that the patient has lived for more than 22 months without anti-leukemia therapy. During that time, she only received anti-HBV drugs, i.e., the combination of ADV (10 mg/d), and ETV (0.5 mg/d) intermittently, which had been discontinued for nearly 3 months due to the pandemic of Corona Virus. Therefore we speculated that the very slow progression of APL may be associated with the treatment with anti-HBV drugs.

**Fig. 2:**
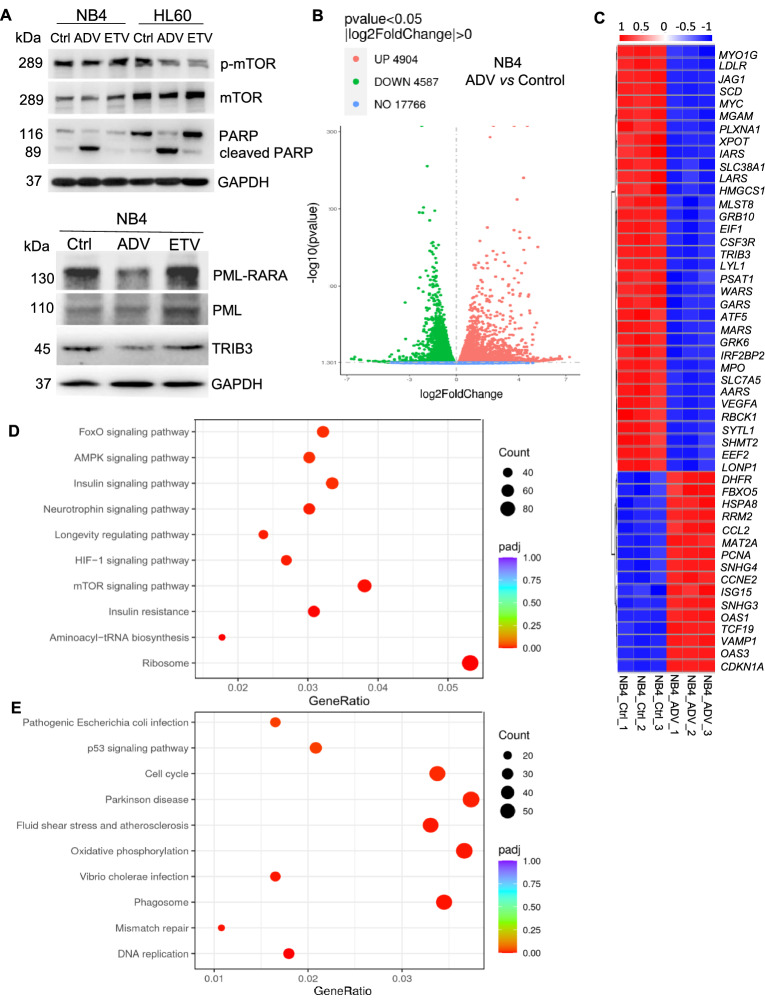
The western blot analysis and RNA-seq results in vitro. **A** Western blot results of PARP, cleaved PARP, mTOR, p-mTOR, PML-RARA, PML, and TRIB3 level in NB4 or HL-60 cells after treatment with 5 µM ADV for 24 h, or 5 µM ETV for 48 h. In NB4 cells, the relative protein density ratio of cleaved PARP to GAPDH in ADV group is much higher than that in control group (7.37:1), and the relative protein density ratio of p-mTOR to GAPDH in ADV group is slightly lower than that in control group (0.79:1). **B** Volcano plots showing differentially expressed genes for NB4 cells after treatment with 5 µM ADV for 24 h. **C** Heat map of TOP50 differentially expressed genes in NB4 cells after treatment with 5 µM ADV for 24 h compared to control (with DMSO only). Each row represents one differentially expressed gene; each column represents one sample. Z-scores are calculated for each row (each gene) and these are plotted instead of the normalized expression values. **D** KEGG pathway analysis of downregulated genes and upregulated genes. Count is the number of DEGs in the given KEGG term, padj is the p-value adjusted for multiple testing, and gene ratio indicates the ratio of the number of DEGs annotated to a KEGG pathway to the total number of DEGs. **E** At 24 h after treatment with 5 µM ADV. RNA-sequencing was conducted in 3 independent experiments with similar results

We next determined whether ADV or ETV contributed to APL inhibition. Both ADV and ETV were nucleoside/ nucleotide analogs, but our results using cell counting kit-8 (CCK-8) assay showed that ADV seemed to have stronger cytotoxic effect than ETV on APL cells (Fig. 1F). A significant increase of apoptosis rates was observed in APL cell line NB4 and AML-M2 cell line HL-60 treated with 5 µM ADV for 24 h analysed with TUNEL assay, but not in counterparts treated with 5 µM ETV (Fig. 1G, Additional file 1: Fig. S1A). Morphological changes of apoptosis were evident with Wright-Giemsa staining (Fig. 1H), and PARP was clearly cleaved in the presence of ADV (Fig. 2A). Thus, ADV showed a greater inhibition of proliferation and induction of apoptosis on APL cells.

Next, the effects of ADV on NB4 and HL-60 cells differentiation were investigated. Increased expression levels of CD11b were observed after treatment with 0.25 µM ADV for 96 h (Fig. 1I). Morphological changes associated with myeloid differentiation were also evident in ADV-treated NB4 cells (Additional file 1: Fig. S1B). These results suggested a differentiation-induction effect of low concentration of ADV on APL cells.

PML-RARA expression of APL blocks promyelocyte differentiation, leading to the proliferation of leukemia cells [1, 3, 4]. In NB4 cells, the oncoprotein PML-RARA was significantly decreased after treatment with 5 µM ADV for 24 h, but no change was observed for PML (Fig. 2A), which is required for the formation of nuclear bodies [5, 6]. However, the level of PML-RARA were not affected in 5 µM ETV-treated cells. These results suggested that ADV could inhibit malignant growth of APL cells through degradation of PML-RARA (but not PML).

To gain insight into the mechanism of ADV’s efficacy in APL, RNA-seq was performed (Additional file 1: Fig. S2). In the 5 µM ADV-treated (24 h) versus vehicle-treated (control) NB4 cells, there were 9491 differentially expressed genes, 4904 upregulated, while 4587 downregulated (Fig. 2B). *TRIB3*, which reportedly could promote rapid APL progression and all-trans retinoic acid resistance by stabilizing PML-RARa and high TWIST1 expression [7, 8], ranked #1 in the list of downregulated genes in ADV-treated versus control NB4 cells (Fig. 2C). In line with this, TRIB3 protein was significantly decreased after treatment with 5 µM ADV (Fig. 2A). Gene ontology analysis revealed a significant decrease in the biological processes of the GO categories “ribosome biogenesis” (Additional file 1: Fig. S1C). Similarly, using KEGG pathway analysis, ribosome protein genes were significantly downregulated by ADV. mTOR signaling pathway was inhibited by ADV via decreasing the phosphorylation of mTOR (Fig. 2D). On the contrary, the p53 signaling pathway, which could be inhibited by increased TRIB3 in APL [8], was activated by ADV (Fig. 2E).

Taken together, the present study demonstrated that ADV exhibited powerful inhibitory effects on APL. More importantly, our study uncovered a novel mechanism of ADV inhibiting APL, which was mediated, at least in part, by inducing cell differentiation and apoptosis via inhibition of TRIB3, and degradation of the oncoprotein PML-RARA.

## Supplementary Information

**Additional file 1.**

## Data Availability

Please contact author for data request.
