# Supplementary material for: Adefovir dipivoxil inhibits APL progression through degradation of the oncoprotein PML-RARA
Source: Exp Hematol Oncol. 2022 Nov 20;11:103. doi: 10.1186/s40164-022-00355-1 (PMC9676767; doi:10.1186/s40164-022-00355-1)

**Adefovir dipivoxil inhibits APL progression through degradation of the oncoprotein PML-RARA**

# Xubo Gong^1*^, Piaoping Kong^1*^, Teng Yu^2^, Xibin Xiao^2^, Lin Wang^1^, Yiwen Sang^1^, Xiang Li^1^, Bin Zhang^3#^, Zhihua Tao^1#^, Weiwei Liu^1#^

**Supplementary Data**

**Supplementary methods**

**Cell lines**

The human APL cell line NB4 and AML-M2 cell line HL-60 were kindly gifted from Dr. Rongzhen Xu (*Cancer Institute, Second Affiliated Hospital, College of Medicine, Zhejiang University, Hangzhou, China*). All cells were maintained in RPMI1640 medium (Gibco) with 10% fetal bovine serum (FBS; Front) at 37°C in 5% CO_2_ and 95% air humidified atmosphere. Primary APL cells were used after patient’ consent. The study was approved by the institutional review board (I20211351).

**Cell Viability Assay**

Cell viability was measured with cell counting Kit-8 (CCK-8; Dojindo Molecular Technologies, Kumamoto, Japan). Briefly, 2×10^4^ cells were seeded in 96-well plate and incubated with or without adefovir dipivoxil (ADV; HY-B0255) or entecavir monohydrate (ETV; HY-13623A), which were purchased from MedChemExpress (MCE, NJ, USA). After 24 or 48 hours, 10 μL of CCK-8 per well was added and incubated in dark at 37 °C incubator for 1 hour. The optical density values were measured at 450 nm using a micro-plate spectrophotometer (BIO-RAD xMark, Hercules, CA, USA).

**Wright-Giemsa Staining**

Wright-Giemsa staining kit was purchased from BASO (Shenzhen). 1100 μL solution A was added and placed for 3 min followed by 500 μL solution B for 1 min. The smear was mildly washed with flowing water and naturally dried. Images were taken by Olympus light microscope (CX31, Japan).

**Assays for apoptosis**

The terminal deoxynucleotidyl transferase-mediated dUTP-biotin nick end labeling (TUNEL) assay was employed to analyze apoptosis. Colorimetric TUNEL apoptosis assay kit was purchased from Beyotime (Item No. C1091, Shanghai, China). Cells were cultured with or without ADV for 24 hours, or ETV for 48 hours. After the incubation, the cells samples were washed with PBS twice, and the cell sediments after low speed centrifugation were collected. Well-made cell smears were immediately fixed with 4% paraformaldehyde for 0.5 hour. The experiments were performed according to the manufacturer’s protocol. Images were taken by Olympus Fluorescence microscope (BX51, Japan).

**Flow cytometry**

NB4 and HL-60 were seeded in 24-well plates (80,000 per well) and treated with 0.25µM ADV for 96 hours. After stained with flow cytometry antibodies (BioLegend, APC anti-human CD11b antibody, Cat# 301310), cells were resuspended in FACS buffer containing 1 µg/mL DAPI and analyzed on Fortessa X20 flow cytometer (BD).

**Western Blot Analysis**

Cell specimens were washed twice with PBS buffer; and total cellular protein was extracted using Radio-Immunoprecipitation Assay buffer (RIPA) containing proteinase and phosphatase inhibitors (Boster, CA, USA) at 4°C for 30 min. Cell extracts were subjected to sodium dodecyl sulfate–polyacrylamide gel electrophoresis (SDS-PAGE), transferred to polyvinylidene difluoride (PVDF) membranes (Millipore, Burlington, MA, USA) and blocked with 5% nonfat milk (Bio-Rad) in TBS–Tween 20 (TBST). The membranes were then reacted with primary antibodies overnight at 4°C. Antibodies against GAPDH were purchased from Cell Signaling Technology (Beverly, MA, USA). Antibodies against PML, mTOR, PARP1, and TRIB3 were purchased from Proteintech (Chicago, IL, USA). Antibodies against PML/RARA were purchased from ABclonal (Wuhan, China). After being washed three times with TBST, membranes were probed with a horseradish peroxidase-conjugated secondary antibody (Millipore, MA, USA) for 1 hour at room temperature. The bound antibodies were visualized using an enhanced chemiluminescence kit (Millipore, Billerica, MA, USA). Western blot images were captured by ChemiDoc XRS+ System (Bio-Rad). All results were repeated independently thrice with triplicate.

**RNA-seq Library Preparation and Sequencing**

RNA integrity was assessed using the RNA Nano 6000 Assay Kit of the Bioanalyzer 2100 system (Agilent Technologies, CA, USA). RNA-sequencing was performed according to the manufacturer’s protocol using the Illumina TruSeq RNA Sample Preparation Kit V2 (San Diego, CA). Briefly, 500 ng of each total RNA sample was used for poly (A) mRNA selection and fragmentation, followed by first and second strand synthesis, end repair, adenylation of the 3′ ends, and adapter ligation. The ligated material was amplified by PCR and then purified with AMPure XP system (Beckman Coulter, Beverly, USA). Library quality was assessed on the Agilent Bioanalyzer 2100 system. Libraries were loaded on an Illumina HiSeq 2500 for parallel sequencing.

**RNA-seq Data Analysis**

Differential expression analysis between groups was performed using the edgeR R package (3.22.5). The P values were adjusted using the Benjamini & Hochberg method. Padj less than 0.05 & |log2(FoldChange)| > 0 were used as the threshold for significantly differential expressed gene. Gene Ontology (GO) enrichment analysis of differentially expressed genes was implemented by the clusterProfiler R package. GO terms with corrected P value less than 0.05 were considered significantly enriched by differential expressed genes. Kyoto Encyclopedia of Genes and Genomes (KEGG) is a database resource generated by genome sequencing and other high-throughput experimental technologies (http://www.genome.jp/kegg/). We used cluster Profiler R package to test the statistical enrichment of differential expression genes in KEGG pathways. We used clusterProfiler R package (3.8.1) to test the statistical enrichment of differentially expressed genes in the Reactome pathway, the DO pathway, and the DisGeNET pathway.

**Statistical Analysis**

Student’s t-test (two-sided) was applied, and changes were considered statistically significant for *P* < 0.05. The data were normally distributed and variation within and between groups was not estimated. Data are presented as mean ± SD (standard deviation of the mean) of at least three biological replicates. Statistical significance was shown as ^*^*p* < 0.05, ^**^*p* < 0.01, and ^***^*p* < 0.001. Statistical analysis was conducted using the GraphPad Prism software packages (La Jolla, CA, USA).

**Supplementary table 1** A panel of 56 genes were tested with RT-PCR in our patient

| BCR-ABL1 P190 | - | MLL-AF4 | - | AML1-MTG16 | - |
| --- | --- | --- | --- | --- | --- |
| BCR-ABL1 P210 | - | MLL-AF6 | - | TEL-AML1 | - |
| BCR-ABL1 P230 | - | MLL-AF10 | - | TCF3-HLF | - |
| PML-RARA L (bcr-1) | - | MLL-ENL | - | MLL-AF5 | - |
| PML-RARA S (bcr-3) | + | MLL-AFX | - | MLL-AF9 | - |
| PML-RARA V (bcr-2) | - | MLL-AF1p | - | MLL-AF17 | - |
| BCR-PDGFRA | - | ETV6-JAK2 | - | MLL-ELL | - |
| ETV6-PDGFRA | - | SIL-TAL1 | - | MLL-AF1q | - |
| KIF5BE-PDGFRA | - | WT1 | - | MLL-SEPT6 | - |
| TEL-PDGFRB | - | CDK5RAP2-PDGFRA | - | ETV6-ABL1 | - |
| CBFβ-MYH11 | - | FIP1L1-PDGFRA | - | CALM-AF10 | - |
| NUMA1-RARα | - | STRNE6-PDGFRA | - | HLXB9-ETV6 | - |
| STAT5b-RARα | - | TLS-ERG | - | NUP98-HoxA9 | - |
| NPM1-RARα | - | AML1-ETO | - | NUP98-HoxA13 | - |
| SET-CAN | - | PRKAR1A-RARα | - | NUP98-HoxD13 | - |
| HOX11 | - | FIP1L1-RARα | - | NUP98-HoxC11 | - |
| HOX11L2 | - | PLZF-RARα | - | NUP98-PMX1 | - |
| NPM1-MLF1 | - | DEK-CAN | - | NUP98-HoxA11 | - |
| TCF3-PBX1 | - | AML1-MDS1-EVI1 | - |  |  |

+，positive；-，negative

**Supplementary figures**

**Figure S1.** The apoptosis of HL-60 cells, the myeloid differentiation of leukemia cells, and the gene ontology analysis of the downregulated target genes induced by ADV. (A) After treatment of 5 μM ADV for 24 hours, it induced the apoptosis of HL-60 cells with TUNEL assay (39.17 ± 1.20% *vs* 1.0 ± 0.29%, *P* < 0.0001). But the apoptosis was not increased in presence of 5 μM ETV for 48 hours compared with control group (cells treated with DMSO alone). Scale bar, 25 μm. (B) After treatment of 0.25 μM ADV for 96 hours, morphological changes associated with myeloid differentiation, including nuclear condensation and lobulation (black arrow), decreased cytoplasmic basophilia (red arrow), were also evident with Wright-Giemsa staining. (C) After treatment with 5 μM ADV for 24 hours compared to control (with DMSO only), gene ontology analysis of the downregulated target genes.

**Figure S2.** The heat map of RNA-seq after treatment with ADV.

RNA-seq was performed using RNA from NB4 cells treated with 5 μM ADV for 24 hours or 5 μM ETV for 48 hours, and HL60 cells treated with 5 μM ADV for 24 hours.

**Supplementary figure 1**


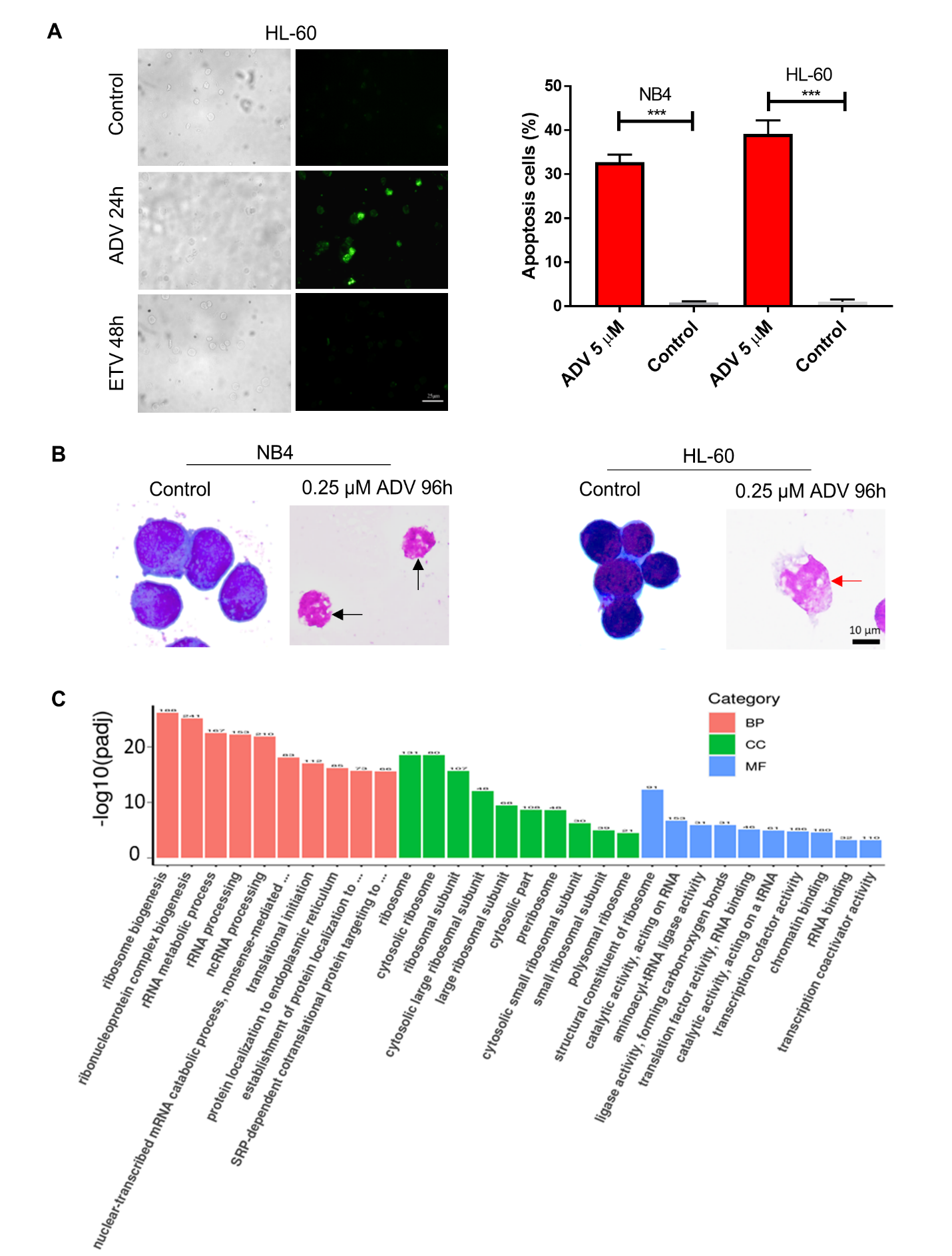


**Supplementary figure 2**


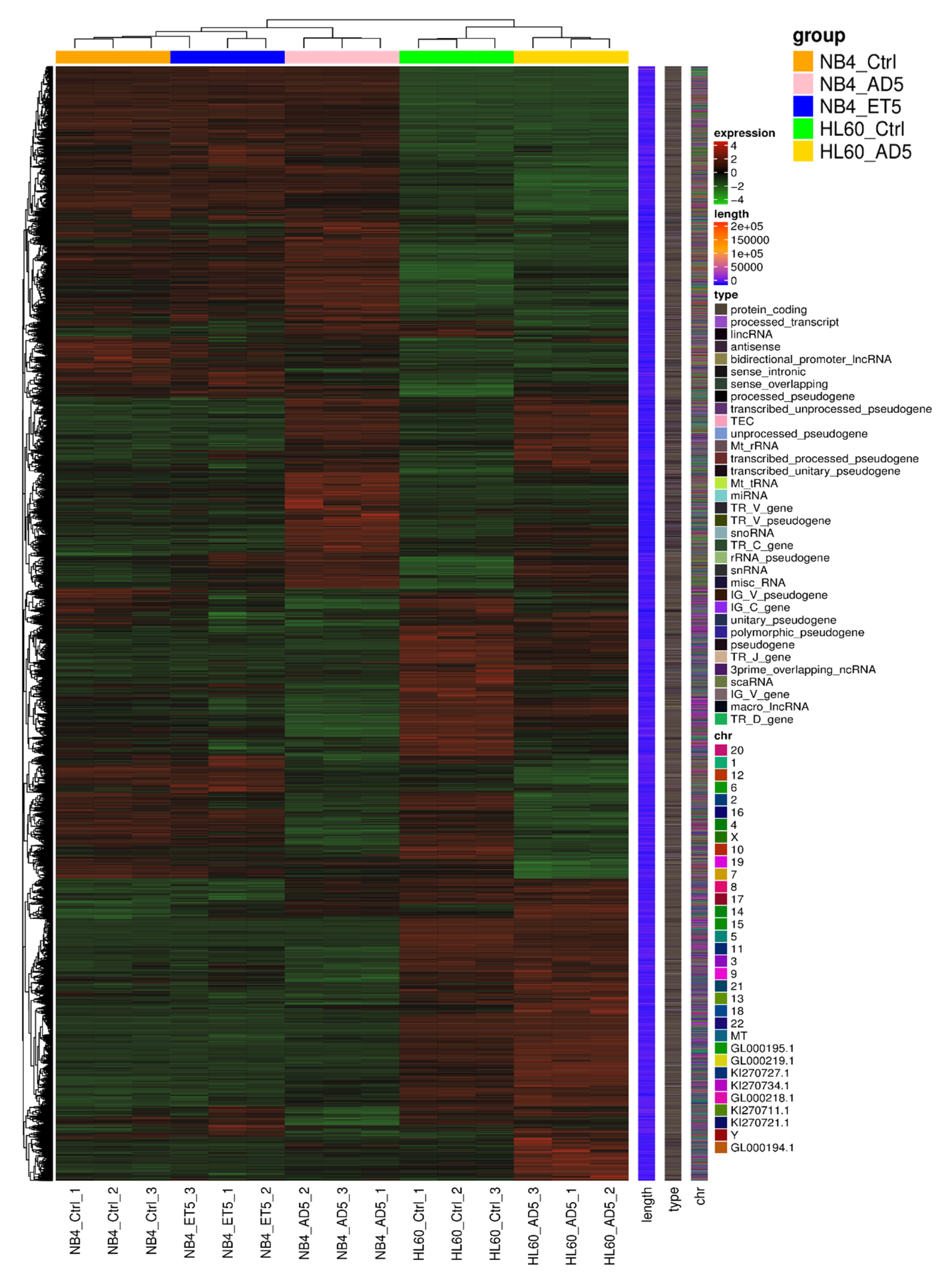

Supplement: Supplementary file 1 — Additional file 1. [file 40164_2022_355_MOESM1_ESM.docx]
